# Injury rates and injury risk factors among federal bureau of investigation new agent trainees

**DOI:** 10.1186/1471-2458-11-920

**Published:** 2011-12-13

**Authors:** Joseph J Knapik, Tyson Grier, Anita Spiess, David I Swedler, Keith G Hauret, Bria Graham, James Yoder, Bruce H Jones

**Affiliations:** 1 U.S. Army Institute of Public Health, Aberdeen Proving Ground, Maryland, USA; 2 Department of Health Policy and Management, Bloomberg School of Public Health, Johns Hopkins University, Baltimore, MD, USA; 3 Federal Bureau of Investigation, Human Resources Division Office of Medical Services, Health Care Programs Unit, Washington, DC, USA

**Keywords:** Physical training, Age, Body mass index, Physical activity, Physical fitness

## Abstract

**Background:**

A one-year prospective examination of injury rates and injury risk factors was conducted in Federal Bureau of Investigation (FBI) new agent training.

**Methods:**

Injury incidents were obtained from medical records and injury compensation forms. Potential injury risk factors were acquired from a lifestyle questionnaire and existing data at the FBI Academy.

**Results:**

A total of 426 men and 105 women participated in the project. Thirty-five percent of men and 42% of women experienced one or more injuries during training. The injury incidence rate was 2.5 and 3.2 injuries/1,000 person-days for men and women, respectively (risk ratio (women/men) = 1.3, 95% confidence interval = 0.9-1.7). The activities most commonly associated with injuries (% of total) were defensive tactics training (58%), physical fitness training (20%), physical fitness testing (5%), and firearms training (3%). Among the men, higher injury risk was associated with older age, slower 300-meter sprint time, slower 1.5-mile run time, lower total points on the physical fitness test (PFT), lower self-rated physical activity, lower frequency of aerobic exercise, a prior upper or lower limb injury, and prior foot or knee pain that limited activity. Among the women higher injury risk was associated with slower 300-meter sprint time, slower 1.5-mile run time, lower total points on the PFT, and prior back pain that limited activity.

**Conclusion:**

The results of this investigation supported those of a previous retrospective investigation emphasizing that lower fitness and self-reported pain limiting activity were associated with higher injury risk among FBI new agents.

## Background

To accomplish its law enforcement and protective missions, the Federal Bureau of Investigation (FBI) trains about 700 new agents each year. New agent training classes at the FBI Academy in Quantico, Virginia, are currently 21-weeks in length. Classes contain a maximum of 50 individuals and new classes begin about every two weeks. During training, new agents are required to participate in a variety of activities which include defensive tactics, practical applications, firearms training, exercise, and classroom/computer activities. Defensive tactics training includes boxing, self-defense, and suspect apprehension techniques. Practical application training consists of driver training, conducting surveillance, room entries, room clearing, and arrest procedures. Firearms' training involves marksmanship, close combat skills, and moving in teams with weapons. To graduate, new agents must pass a Physical Fitness Test (PFT) (sit-ups, push-ups, 300-meter sprint, and 1.5-mile run) and tests are routinely administered at least three times during the new agent training course. While at the FBI Academy, most new agents are expected to perform physical fitness training on their own, although those who failed the first PFT are required to attend group physical training three times per week. In addition to physical tasks, FBI new agents spend considerable time in the classroom involved in lectures and on-line training on law enforcement topics and investigative techniques. The curriculum currently has over 900 hours of training, including 94 hours of defensive tactics training, 95 hours of practical application training, and 114 hours of firearms training.

The physical activities performed by new agents place them at risk of injury. We previously reported on a retrospective investigation of injury rates, physical fitness and the association of these in FBI new agent training [1]. The purpose of the present investigation was to expand on this earlier project by prospectively examining a larger number of potential risk factors in a separate cohort of FBI new agents.

## Methods

This investigation employed a prospective cohort design. On the second day of the FBI new agent training course, individuals completed a PFT. Early on the third day, new agents were briefed on the project and those who consented to participate signed a form and completed a questionnaire. The questionnaire contained items on tobacco use, prior physical activity, prior pain and injuries, menstrual history, date of birth, height, and weight. Injuries during training were tracked from a database at the FBI Academy Health Clinic, as described below. The project took place over the course of a year (March 2009-March 2010) with new agents enrolled in the investigation as new classes started. Participants were followed until they graduated or attrited from training. The FBI's Human Use Review Committee approved the investigation.

### Physical fitness data

PFT scores were obtained from the Physical Training Unit (PTU) of the FBI Academy. The PTU administered the PFT using well-standardized methods. The PFT events were identical for men and women and consisted of four scored events administered in this order: push-ups requiring continuous motion to exhaustion, 1-minute bent-leg sit-ups, a 300-meter sprint, and a 1.5-mile run. Pull-ups to exhaustion were also tested as the last event, but this event was not considered in the total PFT score. At least 5 minutes of rest were provided between events. Gender-specific points were assigned to various levels of performance on each PFT event. To pass the PFT, a point score of 12 was required, with at least 1 point achieved on each event (maximum possible score was 40 points). Details of the PFT events and scoring system are provided on-line [2].

### Final student status

Data on final student status were obtained from a list provided by the Training Director's Office at the Academy. This indicated students who did not graduate, the date they left the course, and the reasons for attrition.

### Injury data

Medical care providers at the FBI Academy Health Clinic at Quantico, Virginia, routinely entered information on medical encounters into a computerized database. Trained personnel examined each new agent medical encounter and determined whether it was for an injury (defined below) or for other medical care. For each injury encounter, information extracted included the date of the visit, the type of visit (new injury visit or follow-up on a previous visit), diagnosis, anatomical location, and activity associated with the injury. In addition to the encounters in the medical database, injury information was also obtained from the United States Department of Labor's CA-1 forms (Federal Employee's Notice of Traumatic Injury and Claim for Continuation of Pay/Compensation). This form provided additional information on the diagnosis, anatomical location, and the activity associated with the injury.

An injury case was defined as a new agent who sustained physical damage to the body [3] and sought medical care or medical compensation one or more times during the FBI new agent training course. Injuries were grouped by "type" as determined from descriptive information in the medical notes and/or CA-1, and by the specific diagnosis. Injury types included 1) overuse injury, 2) traumatic injury, 3) environmental/other injury, and 4) any injury. Overuse injuries were those due to, or related to, long-term repetitive energy exchanges, resulting in cumulative microtrauma. Specific overuse injury diagnoses included tendonitis, bursitis, fasciitis, muscle injury associated with overuse (strain), joint injury associated with overuse (sprain), retropatellar pain syndrome, impingement, degenerative joint conditions, shin splints and musculoskeletal pain (not otherwise specified). Traumatic injuries were those due to sudden energy exchanges (acute event), resulting in abrupt overload with tissue trauma. Specific traumatic injury diagnoses included muscle injury associated with an acute event (strain), joint injury associated with an acute event (sprain), dislocation, fracture, abrasion, laceration, contusions, closed head injury/concussion and pain (associated with an acute event). Environmental/other injuries were those due to exposure to weather, animals, or chemicals and included heat-related injuries, insect bites, chemical exposures, and exertion-related events. "Any injury" included overuse and trauma diagnoses as described above, but excluded environmental/other injuries. The "any injury" type included primarily musculoskeletal injuries, but also included dermatological events (e.g., abrasions, lacerations).

New injuries were first patient encounters resulting in a particular injury diagnosis at a particular anatomical location. Follow-up injuries were subsequent encounters for the same injury. If follow-up visits occurred, they were used in conjunction with the initial encounter to determine the final diagnosis for a specific injury. Thus an initial diagnosis could be changed as a result of a more specific diagnosis at a higher level of medical care.

### Data analysis

All analyses were performed with Predictive Analytic Software, Version 18.0.0. Cumulative injury incidences for any injury, overuse injury, and traumatic injury were calculated as:

(newagentswith≥injury÷totalnumberofnewagents)×100%

Injury incidence rates for any injury, overuse injury, and traumatic injury were calculated as:

(newagentswith≥injury÷totalnumberofnewtraining)×100%

Chi-square statistics were used to make comparisons among men and women on injury incidence; a chi-square for person time was used to compare men and women on injury incidence rates.

Cox regression (survival analysis) was used to examine the association between the time to the first injury (any injury) and independent variables (potential injury risk factors) from the PFT and questionnaire. For each analysis, once a new agent had an injury, his or her contribution to time in training was terminated (censored). Those who attrited from training had their time censored at the day they left training, unless their time had already been censored as the result of an injury. All potential risk factors were entered into the regression models as categorical variables. Continuous variables were converted to quartiles (four groups of approximately equal size) or tertiles (three groups of approximately equal size) based on the distribution of scores. Body mass index (BMI) was also examined in categories according to National Institute of Health Guidelines [4]. Some categories of nominal and ordinal variables were combined to increase statistical power. Because of the relatively narrow age range (24-39 years), age was categorized into only two groups, above and below 30 years of age. For all Cox regressions, simple contrasts were used, comparing the hazard at a baseline level of a variable (defined with a hazard ratio (HR) of 1.00 and called the "referent") with other levels (strata) of the same variable. Univariate Cox regressions established the individual associations between time to first injury at each stratum of a variable. Variables were included in a multivariate backward stepping Cox regression if they achieved *p *< 0.10 in the univariate analyses [5]. Multivariate Cox regressions established the effect of multiple independent risk factors on injury risk.

## Results

There were a total of 531 new agents who enrolled in the project, 426 men and 105 women. Of the 534 new agents who were briefed, three declined to participate. Of the 531 who enrolled, 12% (n = 50) of men and 33% (n = 35) of women did not complete the FBI new agent course. Among the men, injuries and failure to pass the PFT accounted for 26% (n = 13) and 14% (n = 7) of the attrition cases, respectively. Among the women, injuries and failure to pass the PFT accounted for 14% (n = 5) and 3% (n = 1) of the attrition cases, respectively.

### Descriptive data on injuries

Table 1 shows the number and proportion of new injuries and follow-ups by diagnosis. Overuse injuries made up 14% of the new injury cases while traumatic injuries made up 68%. Diagnoses with the largest number of new injury cases were traumatic sprains (joint injuries), traumatic strains (muscle injuries), musculoskeletal pain associated with trauma, abrasions/lacerations, contusions, and insect bites. These six diagnoses accounted for 70% of all new injury cases. Musculoskeletal pain involved medical encounters in which an individual reported pain in a specific musculoskeletal location but no specific diagnosis was provided in the record. The determination that this was a traumatic- or overuse-type injury was based on notes indicating that the complaint was associated with a sudden or acute event (traumatic) or that the pain had occurred gradually over time (overuse).

**Table 1:** Injury cases by diagnoses

Type of Injury		Diagnosis	New Injuries		Follow-Ups	
			
			N	%	N	%
ANY INJURY	OVERUSE	Tendonitis	10	3.2	3	6.8
		
		Bursitis	2	0.6	0	0.0
		
		Retropatellar pain syndrome	5	1.6	2	4.5
		
		Muscle injury (overuse strain)	14	4.4	2	4.5
		
		Neurological	1	0.3	0	0.0
		
		Joint injury (overuse sprain)	4	1.3	1	2.3
		
		Musculoskeletal pain (overuse)	6	1.9	0	0.0
		
		Shin splints	1	0.3	0	0.0
	
	TRAUMATIC	Muscle injury (traumatic strain)	39	12.4	5	11.4
		
		Joint injury (traumatic sprain)	45	14.3	13	29.5
		
		Musculoskeletal pain (traumatic)	37	11.7	3	6.8
		
		Dislocation	7	2.2	0	0.0
		
		Bone Fracture	4	1.3	2	4.5
		
		Tooth Fracture	2	0.6	0	0.0
		
		Nasal Fracture	2	0.6	0	0.0
		
		Abrasion or laceration	33	10.5	2	4.5
		
		Contusion	33	10.5	4	9.1
		
		Closed Head Injury/Concussion	11	3.5	4	9.1

ENVIR/OTHER		General heat-related injury	3	1.0	2	4.5
		
		Exertion	11	3.5	0	0.0
		
		Insect bites or stings	33	10.5	1	2.3
		
		Chemical Burn (capsicum spray)	12	3.8	0	0.0

	Total Cases		315	100.0	44	100.0

The most common anatomical locations for new injuries were the knees (9.8%), thighs (7.7%), shoulders (7.6%), fingers (7.3%), lower back (5.7%), face (5.7%), head (5.1%), and ankles (4.8%). For follow-ups, the most common anatomical locations were the ankle (20.5%), shoulder (13.6%), knees (9.1%), thigh (9.1%), and head (9.1%).

Table 2 shows new injuries and follow-ups by the training activity associated with the injury. Two activities, defensive tactics and physical fitness training, were associated with 78% of the new injury cases and 86% of the follow-up cases.

**Table 2:** New injury cases by associated training activity

Activity	New Injuries		Follow-Ups	
	
	N	%	N	%
Defensive Tactics	184	58.4	25	56.8

Physical Fitness Training	63	20.0	13	29.5

Physical Fitness Testing	15	4.8	2	4.5

Firearms Training	8	2.5	0	0.0

Off-Duty, Academy	1	0.3	0	0.0

Off Duty, Not Academy	6	1.9	0	0.0

Practical Applications Training	5	1.6	1	2.3

Sports	1	0.3	0	0.0

Other	6	1.9	0	0.0

Unknown	26	8.2	3	6.8

Total	315	100.0	44	100.0

### Cumulative injury incidence and injury rates

Table 3 shows cumulative injury incidence and injury incidence rate by gender and injury type. Overall injury incidence (any injury) was slightly higher among the women, the result of a higher incidence of both overuse and traumatic injuries. In consonance with the injury incidence, the overall injury incidence rate (any injury) was slightly higher among the women, accounted for by a higher incidence of both overuse and traumatic injuries. The men had a mean ± standard deviation (SD) of 137 ± 27 days in training while the women had 130 ± 29 days. The total days of training for men were 58,550 and for the women 13,680.

**Table 3:** Cumulative injury incidence and injury incidence rates by gender and injury type

	Injury Type	Men	Women	Risk Ratio - Women/Men (95% Confidence Interval)	p-value^a^
Cumulative Injury Incidence (%)	Any	35.0	41.9	1.20 (0.93-1.55)	0.19
	
	Overuse	5.6	13.3	2.36 (1.27-4.42)	<0.01
	
	Traumatic	31.2	37.1	1.19 (0.90-1.58)	0.25

Injury Incidence Rate (injuries/1,000 person-days)	Any	2.54	3.22	1.26 (0.90-1.77)	0.09
	
	Overuse	0.41	1.02	2.50 (1.29-4.83)	<0.01
	
	Traumatic	2.27	2.85	1.26 (0.88-1.79)	0.11

^a^Chi-square statistic

### Univariate analysis of injury risk factors

Table 4 shows the univariate associations between any injury and age, physical characteristics, and physical fitness. Most women (74%) could not perform pull-ups, so this variable was separated into women who could perform one or more pull-ups and those who could not. There was little association between injury risk and physical characteristics, although men in the third highest quartile of BMI had higher injury risk than those in the first quartile. Among the men, higher injury incidence was associated with older age, slower 300-meter sprint time, slower 1.5-mile run time, and fewer total PFT points. Among the women, higher injury incidence was associated with slower 300-meter sprint time, slower 1.5-mile run time, and fewer total PFT points. Injury risk tended to be elevated for both men and women in the lower quartiles of performance for all physical fitness measures.

**Table 4:** Univariate associations between injury risk^a ^and age, physical characteristics, and physical fitness

Variable	Men					Women				
	
	Strata	N	Injured(%)	Hazard Ratios (95%CI) from Cox Regressions	p-value(from Wald statistic)	Strata	N	Injured(%)	Hazard Ratios(95%CI) fromCox Regressions	p-value(from Waldstatistic)
Age	24.4-29.9 Years	188	26.6	1.00	Referent	24.1-29.9 years	60	36.7	1.00	Referent
	
	30.0-38.6 Years	238	41.6	1.83 (1.30-2.57)	<0.01	30.0-37.0 years	45	48.9	1.52 (0.84-2.75)	0.16

Height	144-175 cm	123	35.0	1.02 (0.69-1.51)	0.93	147-162 cm	39	46.2	1.49 (0.72-3.10)	0.28
	
	176-178 cm	73	30.1	0.79 (0.48-1.28)	0.34	163-168 cm	31	45.2	1.45 (0.67-3.14)	0.35
	
	179-180 cm	65	38.5	1.10 (0.69-1.75)	0.70	169-180 cm	35	34.3	1.00	Referent
	
	181-205 cm	165	35.8	1.00	Referent					

Body Mass	51.8-75.9 kg	109	33.9	1.00	Referent	47.7-56.8 kg	35	42.9	1.00	Referent
	
	76.0-81.8 kg	127	33.1	0.98 (0.61-1.57)	0.91	56.9-63.6 kg	35	45.7	1.15 (0.57-2.33)	0.70
	
	81.9-88.6 kg	99	32.3	0.98 (0.61-1.57)	0.92	63.7-90.9 kg	35	37.1	0.91 (0.43-1.91)	0.80
	
	88.7-113.6 kg	91	41.8	1.24 (0.79-1.95)	0.35					

Body Mass Index	16.51-23.74 kg/m^2^	108	34.3	1.00	Referent	18.25-21.26 kg/m^2^	37	43.2	1.00	Referent
	
	23.75-25.11 kg/m^2^	110	25.5	0.74 (0.46-1.22)	0.24	21.27-23.30 kg/m^2^	34	44.1	1.13 (0.56-2.29)	0.73
	
	25.12-26.63 kg/m^2^	103	48.5	1.54 (1.00-2.36)	0.05	23.31-29.54 kg/m^2^	34	38.2	0.93 (0.45-1.94)	0.85
	
	26.64-38.74 kg/m^2^	105	32.4	0.95 (0.60-1.52)	0.84					

Body Mass Index	16.5-24.99 kg/m^2^	191	30.4	1.00	Referent	16.5-24.99 kg/m^2^	94	42.6	1.00	Referent
	
	25.00-29.99 kg/m^2^	223	36.3	1.18 (0.89-1.92)	0.26	25.00-29.99 kg/m^2^	11	36.4	0.92 (0.33-2.57)	0.87
	
	≥ 30 kg/m^2^	12	8.3	0.24 (0.03-1.72)	0.16	≥ 30 kg/m^2^	0	-----	-----	-----

Push-Ups	1-31 repetitions	104	37.5	1.20 (0.76-1.91)	0.44	0-13 repetitions	36	50.0	1.38 (0.67-2.81)	0.38
	
	32-36 repetitions	112	40.2	1.38 (0.88-2.16)	0.16	14-20 repetitions	35	37.1	0.95 (0.44-2.04)	0.89
	
	37-42 repetitions	106	30.2	0.97 (0.60-1.58)	0.91	21-39 repetitions	34	38.2	1.00	Referent
	
	43-71 repetitions	104	31.7	1.00	Referent					

Sit-Ups	28-40 repetition	97	42.7	1.32 (0.83-2.09)	0.23	26-40 repetition	38	42.1	1.28 (0.61-2.71)	0.52
	
	41-44 repetitions	132	34.0	1.05 (0.65-1.68)	0.84	41-44 repetitions	34	47.1	1.44 (0.68-3.05)	0.34
	
	45-47 repetitions	101	29.5	0.84 (0.53-1.34)	0.46	45-51 repetitions	33	36.4	1.00	Referent
	
	48-59 repetitions	96	35.6	1.00	Referent					

300-Meter Sprint	40-44 seconds	123	28.5	1.00	Referent	46-55 seconds	42	35.7	1.00	Referent
	
	45-46 seconds	132	34.1	1.23 (0.79-1.91)	0.36	56-58 seconds	41	39.0	1.17 (0.58-2.37)	0.66
	
	47-48 seconds	108	39.8	1.48 (0.95-2.32)	0.08	59-62 seconds	22	59.1	2.23 (1.06-4.70)	0.04
	
	49-55 seconds	63	41.3	1.71 (1.03-2.84)	0.04					

1.5-Mile Run	8.18-10.35 min	108	25.9	1.00	Referent	10.05-12.06 min	35	40.0	1.00	Referent
	
	10.36-11.10 min	107	30.8	1.24 (0.75-2.05)	0.41	12.07-12.95 min	35	37.1	0.90 (0.42-1.92)	0.80
	
	11.11-11.64 min	104	37.5	1.60 (0.99-2.61)	0.06	12.96-14.92 min	35	62.9	1.95 (1.00-3.80)	0.05
	
	11.65-15.02 min	106	45.3	2.06 (1.30-3.29)	<0.01					

PhysicalFitnessTest Score	1-11 points	114	43.0	1.73 (1.09-2.75)	0.02	2-10 points	38	47.4	2.04 (1.00-4.14)	0.05
	
	12-14 points	107	30.8	1.15 (0.70-1.91)	0.58	11-14 points	32	43.8	1.77 (0.84-3.71)	0.13
	
	15-18 points	108	36.1	1.35 (0.82-2.17)	0.24	15-27 points	35	34.3	1.00	Referent
	
	19-37 points	97	28.9	1.00	Referent					

Pull-Ups	0-4 repetitions	94	41.5	1.12 (0.72-1.75)	0.62	0 repetitions	78	43.6	1.17 (0.58-2.37)	0.66
	
	5-8 repetitions	138	33.3	0.85 (0.55-1.31)	0.46	1-12 repetitions	27	37.0	1.00	Referent
	
	9-11 repetitions	92	27.2	0.64 (0.39-1.06)	0.08					
	
	12-22 repetitions	100	38.0	1.00	Referent					

^a^Any injury (overuse and traumatic)

Table 5 shows the univariate associations between any injury and the questionnaire variables. Among the men, higher injury risk was associated with having never smoked, a lower self-rating of physical activity, and a lower frequency of aerobic exercise in the past 2 months. Men who reported a prior upper or lower limb injury were at higher injury risk. If the men reported that a limb injury prevented physical activity for at least 1 week, or that they had not returned to normal activity since the injury, the risk of injury was further elevated. Men who reported having foot or knee pain that limited activity were at elevated injury risk; back pain limiting activity also elevated injury risk but less so than foot or knee pain. Few of the questionnaire variables were significantly associated with injury among the women. Back pain that limited activity was associated with elevated injury risk.

**Table 5:** Univariate associations between injury risk^a ^and questionnaire variables

Variable Category	Variable	Strata	Men				Women			
			
			N	Injured(%)	Hazard Ratios(95%CI)from CoxRegressions	p-value(from WaldStatistic)	N	Injured(%)	Hazard Ratios(95%CI) fromCox Regressions	p-value(from WaldStatistic)
Tobacco Use	Smoked 100 Cigarettes in Lifetime	No	352	36.9	1.00	Referent	90	41.1	1.00	Referent
		
		Yes	74	25.7	0.63 (0.40-1.03)	0.07	15	46.7	1.17 (0.52-2.61)	0.71
	
	Age Smoked First Cigarette	NeverSmoked	244	41.4	1.00	Referent	64	35.9	1.00	Referent
		
		6-17 years	116	26.7	0.56 (0.37-0.84)	<0.01	23	52.2	1.65 (0.82-3.32)	0.16
		
		≥18 years	66	25.8	0.56 (0.34-0.94)	0.03	18	50.0	1.39 (0.65-3.01)	0.40
	
	Smoked in Last 30 Days	No	413	34.9	1.00	Referent	102	42.2	1.00	Referent
		
		Yes	13	38.5	1.04 (0.42-2.56)	0.92	3	33.3	0.69 (0.09-4.97)	0.71
	
	Smokeless Tobacco Use Last 30 Days	No	402	35.1	1.00	Referent	104	42.3	1.00	Referent
		
		Yes	24	33.3	0.92 (0.45-1.88)	0.82	1	0.0	-----	-----
	
Physical Activity	Self Rating of Physical Activity	Less Active	6	83.3	4.60 (1.88-11.26)	<0.01	3	66.7	2.18 (0.53-9.06)	0.28
		
		About the same	30	26.7	0.75 (0.37-1.52)	0.42	12	33.3	0.69 (0.25-1.94)	0.48
		
		More Active	390	34.9	1.00	Referent	90	42.2	1.00	Referent
	
	Frequency of Aerobic Exercise in Last 2 Months	≤ 1 time/week	2	100.0	5.96 (1.44-24.69)	0.01	0	---	-----	-----
		
		2-4 times/week	264	31.4	0.75 (0.54-1.03)	0.08	56	44.6	1.14 (0.63-2.07)	0.67
		
		≥5 times/week	160	40.0	1.00	Referent	49	38.8	1.00	Referent
	
	Duration of Aerobic Exercise Last 2 Months	0-30 minutes	84	34.5	0.73 (0.45-1.19)	0.02	9	44.4	0.89 (0.29-2.70)	0.83
		
		31-60 minutes	257	32.3	0.70 (0.48-1.03)	0.07	68	38.2	0.77 (0.40-1.45)	0.40
		
		≥61 minutes	85	43.5	1.00	Referent	28	50.0	1.00	Referent
	
	Frequency of Weight Training in Last 2 Months	≤ 1 time/week	104	40.4	1.40 (0.78-2.52)	0.26	24	41.7	0.90 (0.25-3.28)	0.88
		
		2-4 times/week	275	33.5	1.06 (0.61-1.83)	0.84	74	41.9	0.88 (0.27-2.89)	0.84
		
		≥5 times/week	47	31.9	1.00	Referent	7	42.9	1.00	Referent
	
	Duration of Weight Training in Last 2 Months	0-30 minutes	123	33.3	0.80 (0.49-1.31)	0.38	42	40.5	0.46 (0.19-1.10)	0.08
		
		31-60 minutes	241	34.0	0.78 (0.50-1.22)	0.28	53	37.7	0.38 (0.16-0.90)	0.03
		
		≥61 minutes	62	41.9	1.00	Referent	10	70.0	1.00	Referent
	
	Frequency of Playing Sports Last 2 Months	≤ 1 time/week	376	35.4	0.61 (0.09-4.36)	0.62	91	40.7	0.76 (0.34-1.71)	0.51
		
		2-4 times/week	48	31.3	0.50 (0.07-3.82)	0.51	14	50.0	1.00	Referent
		
		≥5 times/week	2	50.0	1.00	Referent	0	---	-----	-----
	
	Duration of Sports in Last 30 Days	0-30 minutes	253	36.8	1.01 (0.64-1.59)	0.97	59	42.4	0.85 (0.37-1.97)	0.71
		
		31-60 minutes	110	30.0	0.79 (0.46-1.35)	0.39	32	37.5	0.71 (0.28-1.80)	0.48
		
		≥61 minutes	63	36.5	1.00	Referent	14	50.0	1.00	Referent
	
Prior Injury	Prior Lower Limb Injury	No	167	29.3	1.00	Referent	40	42.5	1.00	Referent
		
		Yes	259	38.6	1.45 (1.03-2.04)	0.03	65	41.5	1.07 (0.59-1.97)	0.82
	
	Lower Limb Injury Prevented Normal Physical Activity for ≥1 week	No Injury	167	29.3	1.00	Referent	40	42.5	1.00	Referent
		
		No	41	34.1	1.17 (0.65-2.12)	0.62	10	30.0	0.73 (0.22-2.50)	0.62
		
		Yes	218	39.4	1.51 (1.06-2.14)	0.02	55	43.6	1.14 (0.61-2.12)	0.68
		
										
	
	Returned to Normal Physical Activity Since Lower Limb Injury	No Injury	168	29.8	1.00	Referent	40	42.5	1.00	Referent
		
		No	14	64.3	2.97 (1.46-6.05)	<0.01	4	50.0	1.29 (0.30-5.61)	0.73
		
		Yes	244	36.9	1.35 (0.95-1.91)	0.09	61	41.0	1.06 (0.57-1.96)	0.86
	
	Injured Upper Limb	No	248	31.0	1.00	Referent	73	41.1	1.00	Referent
		
		Yes	178	40.4	1.38 (1.00-1.88)	0.05	32	43.8	1.04 (0.55-1.97)	0.90
	
	Upper Limb Injury Prevented Normal Physical Activity for ≥1 Week	No Injury	248	31.0	1.00	Referent	73	41.1	1.00	Referent
		
		No	34	35.3	1.13 (0.62-2.08)	0.70	9	33.3	0.88 (0.27-2.88)	0.83
		
		Yes	144	41.7	1.43 (1.02-2.00)	0.04	23	47.8	1.10 (0.55-2.20)	0.79
	
	Returned to Normal Physical Activity Since Upper Limb Injury	No Injury	247	30.8	1.00	Referent	73	41.1	1.09 (0.58-2.05)	0.97
		
		No	11	72.7	2.93 (1.41-6.08)	<0.01	1	0.0	-----	-----
		
		Yes	168	38.7	1.31 (0.94-1.83)	0.11	31	45.2	1.00	Referent
	
Pain Limiting Activity	Have Foot Pain Limiting Activity at Times	No	363	32.5	1.00	Referent	81	39.5	1.00	Referent
		
		Yes	63	49.2	1.64 (1.10-2.44)	0.01	24	50.0	1.46 (0.75-2.84)	0.27
	
	Have Knee Pain Limiting Activity at Times	No	313	31.3	1.00	Referent	82	41.5	1.00	Referent
		
		Yes	113	45.1	1.67 (1.19-2.34)	<0.01	23	43.5	1.24 (0.61-2.51)	0.55
	
	Have Back Pain Limiting Activity at Times	No	341	32.8	1.00	Referent	84	35.7	1.00	Referent
		
		Yes	85	43.5	1.33 (0.92-1.93)	0.13	21	66.7	2.33 (1.23-4.40)	<0.01
	
Menstrual History	Age of Menarche	8-11 Years					9	22.2	0.43 (0.10-1.77)	0.24
		
		12-14 Years					76	46.1	1.00	Referent
		
		≥15 Years					19	31.6	0.61 (0.26-1.45)	0.26
	
	Menstrual Periods Last Year	1-10					20	40.0	0.85 (0.40-1.84)	0.69
		
		11-13					84	41.7	1.00	Referent
	
	Gone ≥6 Months without Menstrual Cycle	No					102	40.2	1.00	Referent
		
		Yes					2	100.0	2.84(0.69-11.77)	0.15
	
	Used Birth Control in Past 12 Months	No					47	48.9	1.52 (0.84-2.77)	0.17
		
		Yes					57	35.1	1.00	Referent
	
	Used Hormonal Therapy in Past 12 Months	No					97	41.2	0.88 (0.27-2.85)	0.83
		
		Yes					7	42.9	1.00	Referent
	
	Ever Pregnancy	No					90	40.0	1.00	Referent
		
		Yes					12	41.7	1.02 (0.40-2.61)	0.96

^a^Any injury (overuse and traumatic)

### Multivariate analysis of injury risk factors

Table 6 shows the results of the multivariate backward-stepping Cox regression examining associations between any injury and the risk factors. Among the men, independent risk factors for injuries included older age, slower sprint speed, slower 1.5-mile run time, having not smoked 100 cigarettes in their lifetime, lower self-rating of physical activity, lower and higher frequency of aerobic training, having a prior upper limb injury (especially if that injury did not allow return to normal physical activity), and having knee pain that limited physical activity. Among the women, independent risk factors for injuries included slower 1.5-mile run time and back pain that limited physical activity.

**Table 6:** Multivariate analysis of injury^a ^risk factors

Gender	Variable	Strata	N	Hazard Ratios(95%CI) fromCox Regressions	p-value(from Waldstatistic)
Men	Age	24.4-29.9 years	187	1.00	Referent
		
		30.0-38.6 years	238	1.70 (1.20-2.42)	<0.01
	
	300-Meter Sprint	40-44 seconds	122	1.00	Referent
		
		45-46 seconds	132	1.34 (0.84-2.16)	0.22
		
		47-48 seconds	108	1.84 (1.31-3.00)	0.01
		
		49-55 seconds	63	1.75 (1.01-3.02)	0.05
	
	1.5-Mile Run	8.18-10.35 minutes	108	1.00	Referent
		
		10.36-11.10 minutes	107	1.14 (0.67-1.94)	0.63
		
		11.11-11.64 minutes	104	1.84 (1.11-3.04)	0.02
		
		11.65-15.02 minutes	106	1.95 (1.19-3.20)	<0.01
	
	Smoked 100 Cigarettesin Lifetime	No	351	1.00	Referent
		
		Yes	74	0.42 (0.25-0.71)	<0.01
	
	Self Rating of PhysicalActivity	Less Active	6	6.68 (2.52-17.70)	<0.01
		
		About the Same	30	0.56 (0.27-1.17)	0.12
		
		More Active	389	1.00	Referent
	
	Frequency of AerobicExercise in Last 2 Months	≤ 1 time/week	2	4.58 (0.97-21.71)	0.06
		
		2-4 times/week	263	0.69 (0.49-0.97)	0.03
		
		≥5 times/week	160	1.00	Referent
	
	Returned to NormalPhysical ActivitySince UpperLimb Injury	No Injury	246	1.00	Referent
		
		No	11	3.36 (1.48-7.62)	<0.01
		
		Yes	168	1.41 (1.00-2.00)	0.05
	
	Have KneePain LimitingActivity Sometime	No	313	1.00	Referent
		
		Yes	112	1.84 (1.28-2.64)	<0.01

Women	1.5-Mile Run	10.05-12.06 minutes	34	1.00	Referent
		
		12.07-12.95 minutes	32	1.00 (0.46-2.18)	0.99
		
		12.96-14.92 minutes	32	1.95 (0.97-3.93)	0.06
	
	Have BackPain LimitingActivity Sometime	No	78	1.00	Referent
		
		Yes	20	2.37 (1.27-4.43)	<0.01

^a^Any injury (overuse and traumatic)

## Discussion and conclusion

The project reported here complemented an earlier retrospective investigation [1] of injuries and physical fitness among FBI new agents by prospectively examining injury rates and exploring a greater number of potential injury risk factors. In the previous retrospective investigation [1], the only risk factors for injury examined were physical fitness measures. In the present prospective investigation, the association between injuries and low physical fitness was replicated, and additional potential risk factors were examined including age, physical characteristics, tobacco use, prior physical activity, self-assessed fitness, prior injury, and menstrual history.

The earlier retrospective investigation [1] reported an overall injury incidence (any injury) of 40% for men and 45% for women in the years 2003 through 2008. The present investigation found a slightly lower incidence of injury: 35% for men and 42% for women. More specific injury diagnoses were reported in the present compared to the previous investigation [1]. For example, in the retrospective investigation, traumatic musculoskeletal pain (not otherwise specified) accounted for 27% of the diagnoses while in the present investigation only 12% of the diagnoses were in this category. The higher diagnostic specificity in the present investigation was likely due to the presence of a full-time physician who was on site at the FBI Health Clinic for the entire period and who made many of the diagnoses. In the retrospective investigation, a physician was on-site for only short periods and on-site nurses made most of the diagnoses.

The specific diagnoses (i.e., exclusive of musculoskeletal pain) that comprised the largest number of new injury cases were strains, sprains, contusions, and abrasions/lacerations. These are common injuries in physically active groups of individuals who are involved in running, sports, recreational activities, and military training [6-16]. Less serious traumatic injuries such as abrasions/lacerations and contusions each accounted for 11% of all injuries. This is comparable to the sports literature in which abrasions and lacerations have accounted for 8%-11% of all injuries [9,12,14], and contusions 6%-24% [8,11,13,14]. With regard to overuse injuries, tendonitis accounted for less than 2%-3% of FBI new agent injuries, but in runners, college athletes, and military trainees this injury accounts for 5%-12% [6-8,10,12,15]. There were few cases of more serious traumatic injuries such as fractures, dislocations, and subluxations; these totaled only 4% of all injuries. In studies of runners and collegiate sports athletes, fractures, subluxations, and dislocations have accounted for 3%-13% of all injuries [6,8,9,11-14]. It appears that more serious injuries were less common in FBI new agent training than in many other groups of active individuals.

From an injury-prevention standpoint, the most important information in the medical records was the activity associated with the injury. In some cases, the injury was of the overuse type and had an insidious onset making it difficult to link to a specific event. In most cases, the injury could be linked to a specific training activity, but recording of activities associated with injury was not standardized in the medical records so that pinpointing the injury producing event was difficult. For example, although 58% of the injuries were associated with defensive tactics, often the medical records provided no further information that could have isolated a more specific training activity (e.g., handcuffing, breaking falls, boxing). Given the emphasis in the curriculum on defensive tactics and physical training and the physical demands of those activities, it is reasonable that they were responsible for most of the injuries. Observations of defensive tactics training revealed that a number of safety features were already in place. For example, during boxing new agents wore boxing gloves, headgear, and mouthpieces; during other defensive tactics training they practiced on cushioned mats, which offered some protection during falls and takedowns. Nonetheless, it is sensible that defensive tactics could be further examined to see if additional safety measures might be implemented.

Older male new agents were at significantly higher injury risk and older female new agents tended to be at higher risk. These findings are in consonance with those in military training [16-22] which also show that older individuals are at higher injury risk compared to younger individuals when they perform similar activities. The reason for the higher susceptibility to injury in older individuals may have to do with age-related changes in stem cells, declines in fitness, and/or prior injury history. The ability of resident stem cells to initiate and conduct tissue repair declines with age [23-25]. This could make older individuals more susceptible to overuse-type injuries, in which small microtraumas accumulate over time and repair in the older tissue does not keep pace with these repeated microtraumas. In the present investigation, only about 14% of injuries were of the overuse type, but it is possible that repetitive microtraumas, coupled with slower repair processes, may also weaken tissue to the point where sudden energy exchanges are more likely to cause acute (traumatic) tissue damage. With regard to fitness, aging results in a loss of muscle mass, muscle strength, muscular endurance, aerobic capacity, and flexibility [26,27]. The loss of aerobic capacity and muscular endurance can begin as early as age 25 [27]. These age-related changes reduce absolute fitness levels and may make injuries more likely, since lower fitness has been shown to be consistently related to injury [15,17,18,28-39]. Contrary to this hypothesis however, older age and lower aerobic fitness were found to be independent injury risk factors in the multivariate model. Further, there were low (non-significant) Pearson product-moment correlations between age and performance on all the fitness events (correlations ranged from −0.04 to 0.19). Thus, the lower potential fitness level of the older individuals is not likely to account for the relationship between age and injury. With regard to prior injuries, it is possible that older individuals may be more likely to have experienced prior injuries that may make them more susceptible to future injuries; prior injuries have been shown to be a risk factor for new injuries in many studies [34,40-50]. To examine this hypothesis, self-reported prior injuries were stratified by age, as shown in Table 7. Injuries in training were higher in the older men, regardless of whether or not they reported a prior upper or lower body injury. Results for the women were less clear, presumably because of the greater variability in the smaller sample. Nonetheless, injury-in-training risk still tended to be higher in the older women regardless of whether or not the women had a prior injury. In addition, older age and a prior upper limb injury that interfered with normal activity were both independent risk factors for training-related injuries in the multivariate analysis for men. Thus, the hypothesis that prior injury may make older individuals more susceptible to injuries in training was not supported in these data.

**Table 7:** Injuries in training stratified by self-reported prior injury and age

Gender	Self-ReportedInjuryType	ResponseCategory	24.1-29.9 Year Olds		30.0-38.6 Year Olds		p-value^a^	Risk Ratio-Older/Younger(95%CI)
					
			N	% Injuredin Training	N	% Injuredin Training		
Men	Lower LimbInjury	No ReportedPrior Injury	73	21.9	94	35.1	0.06	1.60(0.96-2.67)
		
		ReportedPrior Injury	115	29.6	144	45.8	<0.01	1.55(1.11-2.16)
	
	Upper LimbInjury	No ReportedInjury	113	23.9	135	37.0	0.03	1.55(1.04-2.30)
		
		ReportedInjury	75	30.7	103	47.6	0.02	1.55(1.04-2.30)

Women	Lower LimbInjury	No ReportedInjury	22	40.9	18	44.4	0.82	1.09(0.52-2.23)
		
		ReportedInjury	38	34.2	27	51.9	0.15	1.52(0.86-2.68)
	
	Upper LimbInjury	No ReportedInjury	45	37.8	28	46.4	0.47	1.23(0.71-2.12)
		
		ReportedInjury	15	33.3	17	52.9	0.27	1.59(0.68-3.69)

^a^Chi-square statistic

Lower levels of physical fitness were generally associated with higher injury risk, in agreement with our previous retrospective investigation [1]. In the present investigation, this was statistically significant only for the 300-meter run and the 1.5-mile run, but injury risk was still higher in the lower performing strata for all the fitness measures. In addition, slower 1.5-mile run time was an independent risk factor for injury in the multivariate analysis for both men and women. Of particular interest was the association between injuries and the total PFT point score. Both men and women who achieved at least 11-12 total PFT points were at lower injury risk than those who scored below 10 total points. Coincidentally, at least 12 points are currently required to "pass" the PFT at the FBI Academy.

The finding that FBI new agents with lower fitness levels were at higher risk of injury agrees well with military basic training studies [15,17,18,28-39]. However, this finding does not agree with most civilian studies [51-58] which generally find the opposite: individuals with higher fitness levels have higher injury incidence, presumably because higher fit individuals spend more time in physical activities that put them at risk. One of the common characteristics of military basic training and FBI new agent training is that individuals perform many physical activities with their fellow trainees. It is possible that the relationship between lower fitness and higher injury risk can be demonstrated in basic training and in new agent training because in these situations, the level and type of physical training are similar among participants. It is more difficult to demonstrate this association in civilian groups because of the greater variety in the levels and types of exercise and other physical activities.

Little association was found between current cigarette smoking and injury risk among the men or women in this investigation but it must be noted that that smoking frequency and amounts smoked were low in this group. Only 13 men and 3 women reported smoking one or more cigarette in the 30 days before new agent training. Also, the reported average amount of smoking was 2 and 6 cigarettes per day for the men and women, respectively. The combination of the small number of smokers and low cigarette dosage may account for the lack of a relationship. Many previous investigations [16-19,59-69] have shown that higher injury risk was associated with cigarette smoking, and that as the amount of smoking increases so does injury risk [16-19,59,63].

The low prevalence of smoking in the present investigation contrasts with studies in the general population and studies of other law enforcement groups. The Centers for Disease Control and Prevention found that in 2008, 26% of 22-44 year old men and 21% of 22-44 year old women in the general US population reported smoking cigarettes. For those with undergraduate degrees, 12% of men and 10% of women reported smoking cigarettes. Studies of male law enforcement officers in various cities find smoking prevalence ranging from 12% to 51% [70-74].

In contrast to cigarette smoking, self-reported smokeless tobacco use among new FBI agents was similar to that of the general US population and Army recruits. Six percent of men (n = 24) and 1% of women (n = 1) reported using smokeless tobacco in the last 30 days. Data from the National Health Interview Survey indicated that in 2000, 6% of 25-44 year old men and 0.3% of women of all ages use smokeless tobacco on all or most days [75]. In US Army Basic Combat Training in 1998, 7% of men and 1% of women reported smokeless tobacco use [31]. In the present prospective investigation, smokeless tobacco use was not associated with injury. This agrees with a previous studies in US Army Basic Combat Training, [31] but smokeless tobacco use has been found to increase injury risk in Norwegian basic training [17] and it is a risk factor for foot blisters [76,77].

Somewhat perplexing was the lower injury risk among men who reported smoking at some point in their lives compared with those who had never smoked. Specifically, there was lower injury risk among men who reported smoking at least 100 cigarettes in their lives and/or those who reported smoking at some age. This is in contrast to studies of Army [78], Marine [79], and Air Force [36] recruits where there was higher injury risk among men who had smoked at least 100 cigarettes in their lives and/or had smoked at some age. One difference in the FBI and military studies was that few FBI new agents were still smokers while a large proportion of military recruits (who are much younger than FBI new agents) continued to smoke up to the time they enter basic training. Many FBI new agents may have smoked in the past but did not continue this behavior. Beside smoking cessation, these individuals may have adopted other favorable health habits that may have reduced injury risk in training. It should also be noted that, contrary to the men in the present study, the women demonstrated a trend indicating lower injury risk among those who had smoked in the past.

Male new trainees who self-rated themselves as being less physically active than their peers, or who reported exercising <= 1 times/week were at increased risk of injury compared to those in the highest activity categories. Women had a similar trend for self-rated physical activity, but no women reported performing aerobic exercise ≤1 time/week. Although sample sizes were small in these less active categories, the findings are similar to that of Nabeel et al. [80] who reported that Minnesota police officers who were less physically active were more likely to report sprains, chronic pain, or back pain. The present data are also in consonance with previous studies of military training that found increased risk of injury among those who self-reported less physical activity relative to peers [16,18,33,34,78,79,81], or a lower frequency of aerobic activity [16,18,78,79]. Physical activity of the proper intensity, frequency, and duration can increase aerobic fitness, muscle strength, and general health, and can reduce body fat [82-86]. Bone mineral density is higher in physically active individuals[60,87-89] and higher bone mineral density has been associated with greater weekly physical activity [88]. These and other factors may assist in reducing susceptibility to injury among more physically active individuals [90].

The American College of Sports Medicine (ACSM) recommends that, to promote and maintain health, 18-65 year old men and women should perform moderately intense aerobic physical activity for at least 30 minutes on 5 days each week or vigorous activity for at least 20 minutes on 3 days each week [91,92]. To improve aerobic fitness (VO_2_max), long-term physical activity should be conducted at intensities between 50% and 90% of VO_2_max [93,94]. Vigorous physical activity that promotes fitness would be most advantageous for FBI new agents because higher levels of fitness are associated with higher levels of occupational performance [95-97]. Data from the Behavioral Risk Factor Surveillance Study indicated that in 2009, 29% of Americans reported that they performed the ACSM-recommended amount of vigorous activity [98]. ACSM also recommends that to promote and maintain good health, activities that increase muscular strength and muscular endurance should be performed on two or more days per week [91]. In the present investigation 95% of the men (n = 405) and 96% of women (n = 101) reported vigorous aerobic exercise at least 3 times per week for at least 16-30 minutes; 78% of men (n = 332) and 89% of women (n = 93) reported vigorous aerobic exercise at least 3 times per week for at least 31-45 minutes. With regard to weight training, 78% of men (n = 332) and 77% of women (n = 81) reported weight training at least twice per week. Thus, a large proportion of new agents reported favorable amounts of physical activity in the 2 months before entering the FBI Academy.

Male FBI new agents who reported a prior upper or lower limb injury were at higher injury risk, especially if the injury prevented normal activity for 1 week, or if they were not able to eventually return to 100% of normal activity. Much of defensive tactics training involved upper body physical activity and those with upper body limitations may be more susceptible to injury during this training. Other studies of military groups [41,42,47], athletes [43-46,48-50,99], and industrial workers [40] have reported an association between prior and current injuries, especially if the prior injury had occurred in the preceding year [44,45,48-50]. Many injuries may be chronic or recurrent, accounting for at least a part of this relationship. It should be noted that the multivariate analysis suggested a "graded" injury risk depending on the reported degree of recovery among those with a previous upper body injury. That is, individuals reporting a less than full recovery had a higher injury risk than those who do report a full recovery, or those reporting no previous injury at all; those who did report a full recovery were still at elevated risk compared to those who do not report a prior injury, but the magnitude of the risk was lower than those not reporting a full recovery.

Both men and women who self-reported foot, knee or back pain that limited activity tended to have elevated injury risk. This was statistically significant for foot and knee pain among the men and back pain among the women, but reported pain in any of these areas tended to increase injury risk. In addition, knee pain that limited activity among the men and back pain that limited activity among the women was independent risk factors for injury. It might be useful to screen prospective new agents who report a history of pain that limits activity.

### Limitations

Injury diagnoses were limited to descriptions in the medical records and the CA-1 s. The medical database at the FBI Academy did not have standardized codes and diagnoses were determined from narrative descriptions. Many of these did not involve diagnostic tests that would have provided more definitive diagnoses. Nonetheless, the injury incidence, injury diagnoses, and anatomic locations of injuries were similar in both the prospective and retrospective investigations, which suggest the findings are reliable. These data provided a representative look at medical encounters and showed that the major types of injuries were strains, sprains, contusions and abrasions/lacerations, which are common injuries in physically active populations [6-16].

Data on tobacco use, physical activity, prior injury, and other questionnaire variables were based on self-reports, which could be subject to recall bias and social desirability bias. Nonetheless, data on physical activity and prior injury supports prior findings in military and other groups. Further, recall periods for many questions were limited to 30-60 days, which has been shown to improve the validity of questionnaire data [100-102]. The small number of tobacco users, although favorable from a health perspective, most likely limited the ability to find associations between tobacco use and injury, if indeed such a relationship exists among FBI new agents.

## Abbreviations

FBI: Federal Bureau of Investigation; PFT: Physical Fitness Test; PFU: Physical Training Unit; PASW: Predictive Analytic Software; HR: Hazard Ratio; CDC: Centers for Disease Control and Prevention; ACSM: American College of Sports Medicine; 95%CI: 95% Confidence Interval.

## Competing interests

The authors declare that they have no competing interests.

## Authors' contributions

JJK--Contributed to project design, data acquisition, data analysis, data interpretation, drafting and revising manuscript; TG--Contributed to data acquisition, data interpretation, revising manuscript; AS--Contributed to data acquisition, data analysis, data interpretation, drafting and revising manuscript; DS--Contributed to data acquisition, data analysis, data interpretation, revising manuscript; KGH--Contributed to project design, data acquisition, data interpretation, revising manuscript; BG--Contributed to data acquisition, data interpretation, revising manuscript; JY--Contributed to project design, data interpretation, revising manuscript; BHJ--Contributed to project design, data interpretation, revising manuscript. All authors read and approved the final manuscript.

## Author Information

The views, opinions, and/or findings contained in this report are those of the authors and should not be construed as official Federal Bureau of Investigation or Department of the Army position, policy or decision, unless so designated by other official documentation. Approved for public release; distribution is unlimited.

## Pre-publication history

The pre-publication history for this paper can be accessed here:

http://www.biomedcentral.com/1471-2458/11/920/prepub
